# Receiving screened donor human milk for their infant supports parental wellbeing: a mixed-methods study

**DOI:** 10.1186/s12884-022-04789-7

**Published:** 2022-05-31

**Authors:** A. Brown, N. Shenker

**Affiliations:** 1 School of Health and Social Care, Swansea University, Singleton Park, Swansea, UK; 2 Centre for Lactation, Infant Feeding and Translation, Swansea University, Swansea, UK; 3 Department of Surgery and Cancer, Imperial College London, London, UK

**Keywords:** Donor human milk, Premature infant, Breastfeeding, Mental health, Infant, Mother, Qualitative research, Wellbeing

## Abstract

**Background:**

Access to donor human milk (DHM) has primarily been based on the health and development outcomes of premature infants but there has been little examination of the broader impact of an infant receiving it upon parental mental health. Breastfeeding and mental health are closely tied with women who experience breastfeeding difficulties or are unable to meet their own breastfeeding goals often experiencing feelings of guilt, sadness and anger, alongside an increased risk of postnatal depression. The aim of the current study was to explore how experience of receiving DHM for their baby affected the wellbeing of parents.

**Methods:**

UK parents of infants aged 0 – 12 months who had received screened DHM from a milk bank (typically on the neonatal unit or in some cases in the community) completed an online questionnaire exploring their experiences. The questionnaire included Likert scale items examining perceived impact upon infant health, own wellbeing and family functioning alongside open-ended questions exploring perceptions of how receiving DHM affected wellbeing.

**Results:**

Almost all of the 107 participants (women = 102) agreed that receiving DHM had a positive impact upon infant health and development, their own mental and physical health, and their family’s wellbeing. Parents felt relieved that their infant was receiving DHM for health reasons but also due to the experience of being listened to, supported and having their infant feeding decisions facilitated. Receiving DHM helped mothers to process some of their emotions at not being able to breastfeed, in part because knowing their baby was being fed gave them the space to focus on recovery and bonding with their baby. Some parents did experience challenges, feeling guilty at receiving DHM, insecure that another woman was able to feed their baby when they could not, or negative reactions from family. Although the impact of receiving DHM upon breastfeeding was not measured, some women who were working to build their own milk supply noted that it helped motivate them to continue.

**Conclusions:**

DHM may play an important role not only in protecting infant health and development but in supporting the mental health and wellbeing of mothers for whom their infant receiving human milk is important.

## Introduction

Data from clinical trials, longitudinal and cohort research, and experimental studies has established that human milk and breastfeeding protects infant and maternal health [1], but is particularly important in protecting the health and development of premature infants. Premature infants who receive human milk are less likely to develop life-threatening conditions such as necrotising enterocolitis [NEC] [2], late-onset sepsis, and have better cognitive development [3]. However, as a result of prematurity, mother-infant separation, and birth trauma, mothers of the smallest and sickest infants can struggle to produce or express enough milk for their baby [4].

For this reason, human milk banks (HMBs) were created to enable other mothers to donate their breastmilk. Donor human milk (DHM) reduces the incidence of NEC [5–7], sepsis [8], and bronchopulmonary dysplasia [9]. Infants fed with DHM rather than infant formula where there is a shortfall of maternal milk also tolerate full feeds more rapidly and leave hospital sooner [10], with likely consequent cost-effectiveness in the hospital setting [11]. While research has typically focused on the health impacts in infants receiving DHM [12] or DHM content and variability [13], increasing data from international sources shows a beneficial effect on maternal breastfeeding rates when DHM is available in the context of optimal lactation support [14–16].

There is a paucity of evidence regarding the experiences of families whose infants receive DHM and its impact on maternal mental health. In the United States of America, an interview study with 30 mothers of late pre-term and term babies who were using supplemental DHM identified that mothers perceived it as better for infant health, which reduced their anxiety [17]. Another study in the USA that interviewed 50 mothers who received human milk via informal mother to mother sharing reported decreased stress and symptoms of postnatal anxiety and depression, again because they felt that DHM protected their baby from illness. Some felt pride at being able to source milk for their baby, although others found the process of receiving and using DHM stressful [18]. In 2019 a service evaluation of the Hearts Milk Bank, a new non-profit HMB in the UK, identified recurrent reports from parents who indicated receiving DHM for their baby had reduced their symptoms of anxiety and depression post-partum [19]. This study sought to add to this literature by conducting a mixed methods study examining the experiences of having received DHM upon parental mental health in a larger sample in the UK.

## Methodology

### Participants

Parents in the UK with a baby aged 0 – 12 months who had received screened DHM from a milk bank participated in this study. Parents could have received DHM via the hospital i.e., whilst on the neonatal unit or paediatric unit, or when at home. This included being discharged home and continuing to receive DHM or after referral to a UK milk bank. In all cases DHM was provided from a human milk bank and free at the point of use to families. Participants did not purchase milk and cannot do so from NHS or non-profit milk banks in the UK.

The 0 – 12 month time period was selected to allow for sufficient participants to have received DHM and be able to reflect on their experiences but to reduce the risk of recall bias due to significant time passing since they received DHM. Further inclusion criteria were parent aged 16 + , able to complete the survey in the English language and able to give informed consent. Parents who received untreated human milk shared directly by other mothers, i.e., through family, social media or milk sharing communities only were excluded from the study.

The decision was made to include both mothers and fathers / partners in the study because often fathers/ partners are instrumental in organising and collecting DHM. They may also be sourcing milk for an adopted infant or be caring solely for their infant if the mother is critically unwell or has died. STROBE guidelines were followed, with concepts from the CHERRIES checklist for online research also applied where relevant, although this is now somewhat dated. The development of social media and the large rise in internet survey use over the last decade as a result of smartphone availability need to be taken into consideration when using the guidance. Full ethical permission for the study was gained from Swansea University Research Ethics Committee. Participants gave informed consent and all aspects of the study were carried out in line with the declaration of Helsinki. All participants gave informed consent prior to completing the survey.

### Measures

Participants completed an online questionnaire containing both closed and open questions, hosted by Qualtrics. The questionnaire included:

- Demographic details (maternal age, education, ethnicity)
- Details of the infant that they received DHM for (age, sex, gestation at birth)
- Current milk feeding status of their infant (breastfeeding duration and exclusivity, formula use, DHM use, reasons for stopping breastfeeding if relevant)
- Details of DHM use (duration, exclusivity, approximate volume received, milk bank used)
- 5-point likert scale items examining perceived impact of DHM upon infant health and development, own wellbeing and family wellbeing [Strongly agree – strongly disagree]
- Open-ended questions exploring experience of receiving DHM and perceived impacts upon infant health and development, own wellbeing and family wellbeing (Table 1)

**Table 1:** Open-ended survey questions exploring experiences of receiving donor human milk

• Can you tell us a little about what motivated you to want donor milk for your baby?
• How did receiving donor milk make you feel?
• If you were also breastfeeding your baby, how did receiving donor milk affect your experience of breastfeeding?
• What impact do you feel that donor milk had for your baby?
• What impact do you feel that receiving donor milk had for you and your wider family?
• How did your experience of donor milk fit with your expectations of receiving donor milk? Was it better? Different? More challenging than expected?
• Finally, do you have any further comments on the experience of receiving donor milk?

Survey items were designed based on existing themes in the literature, such as perceived impacts upon infant health, maternal health and family functioning. Open-ended questions were constructed to be ‘open’ in nature, i.e., asking participants how receiving DHM made them feel rather than asking ‘did receiving DHM improve your mental health’. For questions that required precise answers such as volume of milk received or which milk bank DHM was received from participants were encouraged to leave questions empty or write ‘not sure’ if they did not know the answer. The survey was reviewed and piloted by staff at a milk bank, consultants in human milk banking, and by parent representatives for clarity. Minor amendments were incorporated into the final survey.

### Procedure

Data were collected for six months from June – December 2020. Advertisements containing brief details of the study and inclusion criteria were placed on social media with encouragement for breastfeeding, milk banking and parenting organisations to share the post. Posts were shared on the academic / organisational pages of the research team including Instagram, Facebook pages and Twitter with encouragement for interested viewers and organisations to share further. Given the wide following of the research teams social media pages (a combined follower total of 100,000 + followers from varied demographic backgrounds) paid targeted adverts were not used.

During the study period the advert was shared over 350 times across social media platforms (with further sharing that could not be tracked for privacy reasons), with a post reach of at least 150,000 accounts. Analytical data from the three platforms estimated that approximately 1200 people who viewed the post engaged with it through clicking for further information, commenting or sharing the post details. This would include eligible participants and individuals interested in the topic and sharing the study advert. Organisations/ individuals that shared the adverts included the UK Association for Milk Banking (UKAMB), individual UK milk banks, breastfeeding charities, the UK World Breastfeeding Trends Initiative, infant feeding teams and health professionals and parents.

Advertisements contained a link to the study information sheet for further information followed by consent questions if interested participants wanted to continue. UK residence was confirmed by participants having a UK postcode. Only when all consent items were agreed did the full questionnaire load. Once completed a debrief statement was given, explaining the study, thanking them for participation, and giving them contact details for support organisations if needed.

### Data analysis

Quantitative data were analysed using SPSS version 27 to compute descriptive statistics regarding perceptions and experience of DHM use. Data directly from the question ‘Can you tell us a little about what motivated you to want donor milk for your baby?’ was mainly used to code the reason for seeking DHM. However, insights from other question responses were also added such as mention of a health issue leading to DHM use. Participants could give more than one reason (i.e., infant prematurity plus a maternal health issue).

A thematic analysis was conducted on qualitative data from the open-ended questions using a simple descriptive analysis [20]. The first author immersed themselves in the data, reading through responses from each participant and across questions for all participants. Next responses were read and re-read to identify smaller themes. These smaller themes were then grouped into larger subthemes [21]. To enhance trustworthiness of the data [22], initial coding was completed by the first author, with the second author reviewing proposed themes and subthemes. Where disagreement occurred, themes were discussed until agreed. An example of the overall process of how the analysis moved from raw data to subtheme, to theme is shown in Table 2.

**Table 2:** Showing analysis process from raw data to theme

Raw data	Subtheme	Subtheme description	Theme	Theme description
‘It’s hard to put in words how it felt to know I could still provide breast milk for the baby…it felt like an army of invisible mothers looking after us and felt very emotional and still does when I think back to it.’	Feeling connected to other mothers	Women felt supported by and connected to a network of other women who had been kind enough to donate their milk to strangers	Feeling supported and care for	The experience of receiving DHM from a milk bank helped mothers feel that someone was listening to them, seeing their desire to give their baby DHM and supporting them

## Results

One hundred and seven participants completed the survey including 102 mothers and five fathers. All participants remained in the study if they completed the Likert scales examining their perceived impact of DHM. However, for the open-ended questions some participants [*n* = 5] gave short responses (providing only a few words over all open-ended questions), wrote that they were ‘unsure’ [*n* = 4] or left the open-ended questions blank [*n* = 3]. This left 95 participants (92 mothers and 3 fathers) who contributed to the qualitative analysis. Responses given were typically in depth and often crossed these themes and subthemes within one response (see Table 3 for a typical example response).

**Table 3:**
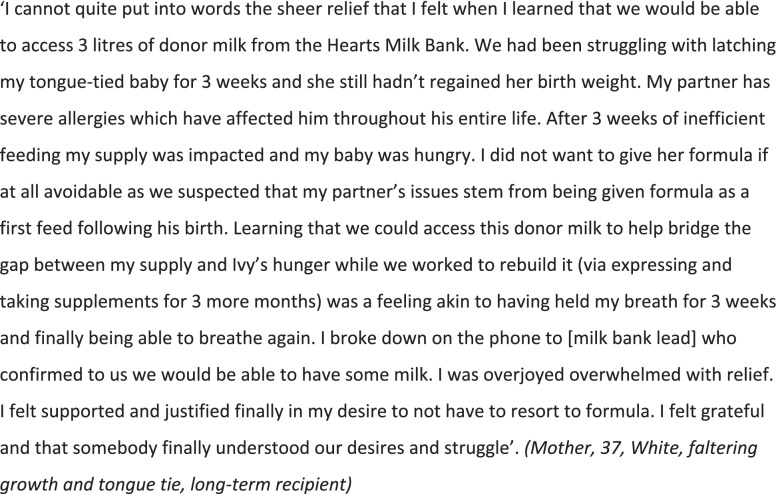
Example of typical depth and length of open-ended response

Mean age of participants was 32.38 (SD: 5.80) with a range from 19 to 47 years. DHM was received for 115 infants (eight sets of twins) of which 48 (44.8%) were male. Age of infant at birth range from 24 to 43 weeks with a mean gestation of 33.1 weeks (SD: 4.64 weeks). Further demographic details are shown in Table 4.

**Table 4:** Participant demographic background

Indicator	Group	N	%
Age (yr)	< 19	2	1.8
	20 – 24	9	8.5
	25 – 29	21	21.4
	30 – 34	32	28.1
	> 35	43	40.2
Education	School	9	8.6
	College	22	20.6
	Higher	52	48.6
	Postgraduate	24	22.4
Marital status	Married Cohabiting Single Widowed	61 40 5 1	57.0 37.4 4.7 0.9
Ethnicity	White	91	85.0
	Gypsy / Traveller / Roma	1	0.9
	Asian or Asian British (Bangladeshi, Indian, Pakistani)	4	3.8
	Asian or Asian British (Chinese) Black or Black British Mixed or multiple Other	2 3 5 1	1.9 2.7 4.7 0.9
Parity	First baby / first multiple birth	59	55.7
	Second or more	48	44.3
Country	England	86	80.4
	Scotland	17	15.9
	Wales	0	0.0
	Northern Ireland	4	3.7
Infant gestation at birth	Extremely preterm (less than 28 weeks)	16	14.9
	Very preterm (28 – 32 weeks)	61	57.0
	Moderate to late preterm (32 – 37 weeks)	21	19.6
	Term (37 weeks plus)	9	8.0

### Receiving donor human milk

Milk was received from nine different HMBs, including seven in England, one in Scotland and one in Northern Ireland (with 8.4% (*n* = 9 participants) not sure which milk bank provided their DHM) (Table 5). For NHS milk banks, participants predominantly received milk from their local milk bank, with participants therefore based across the UK. Almost half the respondents stated that they received milk from ‘Hearts Milk Bank’ which is the only milk bank outside of the NHS in the UK. The Hearts Milk Bank provides milk to 47 hospitals around the UK, alongside families (n = 375) with clinical need in communities across England and Wales, and therefore has a wide geographical reach. On examining the postcode of participants who received milk from Hearts Milk Bank, a large geographical variation in location was seen. The most frequent locations that participants lived in included London, Hertfordshire, Hampshire, Worcestershire, Cambridge, and Oxford.

**Table 5:** Milk bank locations from which milk was received

Milk bank	N	%
Hearts Milk Bank	46	42.9
Milk Bank Scotland	17	15.8
The Milk Bank at Chester	13	12.1
John Radcliffe Hospital Oxford	7	6.5
Queen Charlotte’s and Chelsea Hospital—London	4	3.7
The Southwest Neonatal Network Donor Milk Bank—Bristol	4	3.7
Western Trust Milk Bank Northern Ireland	4	3.7
Kings College Hospital London	3	2.8
Unsure	9	8.4

The majority of infants started receiving DHM in the first days of life. Overall, 35 infants (32.7%) received it from their day of birth, 40 (56.1%) by day three, and 95 (88.8%) by the end of the first week. However, age at starting DHM ranged from birth to 4 months old.

Participants were asked the duration that their infant received DHM exclusively (receiving only DHM feeds) and non-exclusively (DHM alongside own milk or formula). The majority of infants received exclusive DHM at first, but for less than a week (*n* = 90, 84.1%). The majority of infants then went on to receive DHM alongside other milk for up to a week (*n* = 91, 85.0%). However, some infants received DHM for an extended period (Table 6).

**Table 6:** Duration of DHM received

Duration	Exclusive DHM		Alongside other milk	
	**N**	**%**	**N**	**%**
0 days	17	15.9	0	0.0
1—7 days	69	64.5	81	75.7
8 – 14 days	6	5.4	9	8.4
15 – 21 days	8	7.4	7	6.5
22 – 28 days	3	2.7	5	4.6
29—35 days	0	0.0	2	1.9
36 – 42 days	2	1.9	1	0.9
43 – 49 days	1	0.9	1	0.9
50 – 56 days	0	0.0	0	0.0
57 – 63 days	0	0.0	0	0.0
64 – 70 days	1	0.9	1	0.9

When asked to estimate how much donor milk they received overall 59.8% (*n* = 64) provided an estimate with the remainder unsure. This ranged from less than 100 ml spread across supplemental feeds to up to 70 L. However, the majority of infants (*n* = 50, 76.6%) received 5 L or less.

Reasons for receiving DHM are shown in Table 7 and included infant prematurity, low milk supply, maternal illness/medication and maternal cancer / mastectomy. Participants sometimes gave more than one reason meaning percentages in Table 7 add up to more than 100%. Some participants did not give a reason why they needed to use DHM and instead simply gave reasons for why they wanted to use DHM (e.g., to protect their baby’s health). These participants were coded as ‘no reason given’.

**Table 7:** Reasons for requesting DHM

Reason for requesting DHM	N	%
Infant prematurity	81	85.2
Maternal low / absent milk supply	67	62.6
Faltering growth	38	40.0
Supporting breastfeeding difficulties / tongue tie	15	15.7
Maternal cancer /mastectomy	12	9.3
Maternal medication use / health issue	11	10.2
Maternal mammary hypoplasia	6	5.6
No reason given	11	11.5

Participants could select more than one reason, meaning percentages added up to > 100%

### Perceived impact of donor milk

Participants were asked to rate the impact they felt receiving DHM had upon their infant and family (5-point likert scale from very positive to very negative). Participants perceived a very positive or positive impact on their baby for infant health (98.1%, *n* = 104), infant development (96.2%, *n* = 101), own mental health (96.2%, *n* = 101), own physical health (86.8%, *n* = 92) and family’s wellbeing (89.6%, *n* = 85). One participant in each case rated each aspect as having a negative impact with the remainder neutral.

### Exploring experiences of receiving donor milk

Participants were asked a series of open-ended questions exploring their experiences of receiving DHM. A thematic analysis identified seven themes, consisting of 16 subthemes overall. Examples of quotes are given below, with details of participant: parent, age, ethnicity, and where provided details of breastfeeding complication. Participants are further categorised into ‘short-term recipients’ (DHM received for a week or less) and ‘long-term recipients’ (DHM received from more than one week).

## Theme one: Relief that their baby was receiving DHM for health reasons

A central theme to emerge was the perceived impact parents felt that receiving DHM had on their infant’s health and development and the subsequent impact that this had upon parental wellbeing. Parents believed that their infant’s immune system was supported, alongside reassurance for those who were worried about family history of allergy.

‘It brings me great comfort knowing that I was optimising the amount of breastmilk she received. I genuinely believe that her immune system is great because she received different sources of antibodies on top of the antibodies I gave her.’ (Mother, 28, White, Low milk supply, long-term recipient)

‘We have so many allergies between us and we were worried about her being so tiny and the effect that formula might have so we were very relieved to be able to supplement with DHM instead of formula. It helped reduce our anxiety and stress at a difficult time.’ (Father, 42, White, Premature baby, long-term recipient).

## Theme two: Reducing the pressure in the early days

Many participants talked about how receiving DHM made the intense days after a birth (which was often premature or traumatic) easier. DHM reduced pressure regarding providing expressed milk, increased bonding and supported family relationships.

### Reducing pressure to provide expressed milk / build supply

Some women in the study were experiencing breastmilk supply difficulties and needed to supplement. Often women in this situation were trying to express milk for a premature baby who could not feed directly or were on a triple feeding plan whereby they breastfed their baby, supplemented their baby with previously expressed milk and then tried to express more milk again. Although this can help build milk supply more quickly, it can feel exhausting for the mother. Receiving DHM in these circumstances helped reduce the pressure on mothers as they knew that their baby would be fed even if expressing did not go well that day.

‘I was so exhausted from trying to care for her and feed her and express. Just those few feeds of donor milk meant the pressure reduced and I could have a break from the ward for a bit. I think it made all the difference as allowed my stress to come down and for me to catch up on a bit of sleep. I think without that I might have given up breastfeeding altogether but that space, that physical and emotional space that donor milk allowed probably is a big part of why we’re still feeding now.’ (Mother, 32, White, Premature baby, short-term recipient).

### Enabling bonding

Linked to the reduction in pressure to produce sufficient breastmilk for their baby, some mothers described how this enabled them to take a step back and start to properly bond with their baby. It removed the stress from the relationship and feeding experience, allowing more positive interactions with their baby that weren’t focussed on attempting to produce more milk.

‘I think it helped us to bond better. Once I was less stressed and obsessed with making sure he just had my milk it felt as if everything eased between us?’ (Mother, 34, Asian Chinese, Premature baby, short-term recipient)

### Supporting family relationships

Related to taking the pressure off needing to provide sufficient breastmilk themselves, the consequence of this reduced stress and pressure rippled out into the broader experience of early parenting, the transition to motherhood / fatherhood and family relationships. Mothers in particular felt that they could focus some of their attention on other children and felt less tense around their partner.

‘Reduced stress for all family as I was not stressing about not being able to provide the milk’. (Mother, 33, White, Insufficient glandular tissue, long-term recipient)

‘I was a better mum for it, my husband benefitted from this and we felt stronger and united in the parenting journey’. (Mother, 47, White, HIV positive, long-term recipient)

### Enabling partners to play a role

Some mothers accessed DHM direct from the hospital when they were staying on the neonatal unit. However, others sourced DHM from milk banks who were able to provide some DHM to the community. In these cases DHM needed to be organised and collected. Although this could sometimes add an additional layer of stress, a number of parents in the study highlighted how it enabled the father / partner to be able to practically do something and feel like they were contributing to their baby being fed.

‘I was able to go and collect the milk for our baby and that really helped me feel like I was doing something and that I was able to look after my partner in a way because she was very distraught. It also gave me something practical to think about and do rather than worrying about them both all the time.’ (Father, 38, White. Premature baby, long-term recipient).

## Theme three: Feeling supported and cared for

The process of receiving DHM did not only affect parents in relation to feelings around how their baby was fed, but also helped them to feel cared for and supported, often at a very challenging point in time. Parents experienced this in a number of ways:

### Feeling listened to

Many women talked about feeling listened to and respected in relation to how they wanted to feed their baby. It wasn’t just that they were given DHM but that someone asked them questions, listened to their story and saw how much they valued giving their baby DHM.

‘I expected to be laughed at to be honest but the woman I spoke to on the phone was just amazing. She was so kind and listened to how I felt and was so understanding and it made a real difference to my concerns.’ (Mother, 41, White, Low milk supply, long-term recipient).

### Feeling cared for

Parents also felt cared for at a difficult time. Typically, families were in late pregnancy or the early days of having a baby when they were told they needed to supplement their baby or would not be able to breastfeed. They already felt vulnerable and exhausted, yet someone was there helping and caring for them.

‘I felt supported and like someone cared for me and my baby following diagnosed low supply.’ (Mother, 36, White, Insufficient glandular tissue, long-term recipient)

### Feeling supported by and connected to other mothers

Others felt supported by a wider community of breastfeeding mothers who were donating their milk to strangers. Women felt supported by people they had never met and overwhelmed that someone they did not know could be so kind.

‘It’s hard to put in words how it felt to know I could still provide breast milk for the baby...it felt like an army of invisible mothers looking after us and felt very emotional and still does when I think back to it.’ (Mother, 37, White, Double mastectomy, long-term recipient)

Notably, this feeling of connection and gratitude to other women helped encourage some mothers to carry on expressing their own milk and trying to build their supply. They felt that others had stepped up to support them without even knowing them and that they owed it to the community to keep trying.

‘I found the triple feeding in particular really difficult but then I thought about the women who had taken the time to donate their milk and all that entails and it motivated me to keep trying. It felt like they were supporting me in this even though we had never met.’ (Mother, 26, Black British, Faltering growth, short-term recipient)

Some mothers in the study then talked about the circle of going on to provide DHM themselves, feeling grateful of the opportunity to help others in their situation and that they were ‘paying back’ the kindness of others.

‘As soon as we were settled and feeding well I started to build up a stash of my own milk to donate back and it made me feel so good knowing it would likely go to a mum just like me who was stressed and desperate for help and I could play a part in changing all of that.’ (Mother, 28, Black, Premature baby, short-term recipient)

## Theme four: Supporting preferred feeding choices

Another central theme was the impact that receiving DHM had upon mothers’ (and sometimes partners’) feeding plans and preferences. By the nature of this sample breastfeeding and receiving human milk was obviously very important to parents and having the option to be able to give their baby DHM played an important role in wellbeing, eliciting a whole host of emotions. These emotions typically split two ways: emotions at being able to provide DHM and emotions at being able to avoid giving formula milk.

### Opportunity to use DHM

The positive emotional experience of having the opportunity to give their baby DHM, sometimes enabling their baby to have a fully human milk diet, or sometimes in combination with formula milk, was clear. Women used words such as feeling ‘*grateful’*, ‘*relieved’*, ‘*proud’* and ‘*empowered’* at being able to give their baby DHM.

‘It reduced any complicated feelings of guilt, it provided peace of mind, it made me feel happy and proud’. (Mother, 35, White, Double mastectomy, long-term recipient)

The strength and depth of this relief was clear from many of the responses. Women did not simply feel a bit happier, but rather that their mental health was protected.

‘I was devastated when I realised I wasn’t going to produce enough milk myself for her and this felt like a genuine lifeline, that someone was pulling me out of a very dark place. I will always be so grateful to the support and kindness we received.’ (Mother, 29, Asian British Pakistani, Low milk supply, short-term recipient)

### Opportunity to avoid giving formula milk

Conversely many women described their relief and gratitude at not needing to use formula milk when this was not part of their feeding plan.

‘I can say my daughter has been exclusively breastfed - my son had about 40 ml of formula when he was born then breastfed until 3 but in my head I could never say he was exclusively breastfed all the way through and that stuck with me. I didn’t want that again. My values are very much against using formula if there is human milk available and I am so glad that we were able to access this. I would have been so unhappy if that first week had been formula top ups every 3 hours as it just a would have felt to me like putting something in my baby which I don’t believe in. ’ (Mother, 34, Low milk supply, short-term recipient)

### Conflicted and mixed emotions

However, although DHM allowed parents to feed their baby in the way they wanted, many did feel conflicted over using it. This was either in relation to not being able to exclusively breastfeed themselves, or feeling torn at another mother providing the milk for their baby. This sometimes compounded feelings of guilt that they weren’t providing milk for their baby directly themselves.

‘I felt guilty that it wasn’t my milk being given but grateful to be able to give breast milk rather than formula.’ (Mother, 28, White, Premature baby, short-term recipient)

### Guilt at receiving DHM

One challenging emotion experienced amongst some mothers was the guilt at receiving DHM for their baby, believing that it might be taken away from a more vulnerable baby. Some felt that they needed to justify why they were using it to others around them and tell people that they were trying to increase their milk supply to feel deserving of receiving it.

‘ [I felt] guilty for taking donor milk for my baby when more needy prem babies might miss out because we took the milk. I felt a constant need to explain to everyone all the things I was trying to get my supply up after my milk didn’t come in to justify having some donor milk to keep us going’. (Mother, 36, White, Tongue tie & latch issues, short-term recipient)

## Theme five: Alleviating complex emotions at not being able to breastfeed

Some mothers in the survey were using DHM to top up their own milk supply, sometimes due to having insufficient glandular tissue and therefore having a reduced supply or whilst waiting for their own milk supply to fully develop. Others were unable to breastfeed for health reasons and had sourced typically a small amount of DHM for their baby for the early weeks of life. The depth of emotion about how women felt at not being able to breastfeed or needing to supplement their baby was clear. However, receiving DHM supported maternal mental health in a number of ways.

### Reducing feelings of guilt

Many women talked about the guilt they felt at not being able to breastfeed their baby even though in every case described there was a physiological reason why women could not produce sufficient milk or breastfeeding was contraindicated.

‘[I felt] so grateful that I could give my baby the best start in life, humbled that women would donate their precious milk to people like me and less guilty for not being able to breast feed my baby’. (Mother, 35, White, Breast cancer, long-term recipient)

‘It took away a lot of the guilt my wife felt at not being able to fully breastfeed him and that really helped her.’ (Father, 40, White, Partner insufficient glandular tissue, long-term recipient)

### Reducing feelings of failure

Others described feeling like they had failed as a mother in not being able to breastfeed their baby, yet receiving DHM helped them process this belief, feeling like they were supporting their baby by being able to get them DHM. Again, this emotion was common even when women often had no choice due to complex health reasons.

‘I had the deepest urge to breastfeed my baby and I just couldn’t, my milk did not come in, no one can say why, receiving the donor milk made me feel like I wasn’t failing my baby by only formula feeding him.’ (Mother, 39, White, Absent milk supply, long-term recipient)

### Helping women to process their situation

Many of the women in the study had health complications that prevented them from being able to breastfeed. Some had not discovered these until after the birth or had not been told that they wouldn’t be able to breastfeed until late in their pregnancy. The shock at not being able to feed their baby in the way they had hoped for and planned was clear with women finding it difficult to process their feelings about feeding their baby in a different way or how that fitted with their assumptions of how they would care for their baby. DHM appeared to play a role in reducing this.

‘Mentally helped me to come to terms with possibly having insufficient glandular tissue and prepared me for possibly not being to fully breast feed.’ (Mother, 33, White, low milk supply, long-term recipient)

## Theme six: The emotional protection of being able to continue breastfeeding

Looking specifically at mothers who were using DHM as a supplement whilst trying to establish their own milk supply, a common feeling was that using DHM in this way helped them to establish and continue breastfeeding. Many attributed this as helping them to continue as it felt beneficial to keep persevering when things were difficult as their baby had an exclusive human milk diet.

‘She started putting on weight for the first time and moved from the 1st percentile to a healthier level of 9th percentile. It gave her the boost she needed to grow and breastfeed more efficiently, increasing my supply adequately. She is now a healthy 75th percentile baby and she is still exclusively breastfed while exploring solid foods.’ (Mother, 36, Insufficient glandular tissue, long-term recipient).

‘Donor milk saved our breastfeeding relationship. Breastfeeding was so important to me for so many reasons and being able to breathe, knowing my babies had human milk to drink, I was able to undo the damage done by the hospital postnatal experience and relax into relactating for my twins’. (Mother, 38, White, Premature twins, long-term recipient)

The psychological benefits this offered women were significant. Many talked about DHM allowing them to continue with their feeding desires and plans and image of how they would care for their baby, describing the positive impacts of this many months after receiving DHM.

‘I look back now to those early days and marvel at how far we have come. Those few feeds of donated milk gave me the strength to carry on with that punishing triple feeding routine and I am so, so grateful to be in this place still breastfeeding her now at 9 months. She’s had some health challenges due to her prematurity but I feel that I’m doing the best I can for her by breastfeeding her and to be honest I think that some days that’s all that’s preventing me from feeling like I failed her.’ [Mother, 37, Black, Premature baby, short-term recipient]

## Theme seven: The impact of reactions of family and friends

Finally, some parents in the study talked about how the reactions of their friends and family when they heard that they were using DHM affected their wellbeing. For some this was positive, but for others criticism or judgement made things more difficult.

### Positive, supportive reactions

Some parents in the study experienced positive reactions from friends and family who were eager to find out more about DHM or had positive knowledge about the impact it could have.

‘I felt that in a very difficult circumstance (cancer treatment 3 weeks post delivery) I was heard, supported, encouraged. My family (parents) were relieved as well’. (Mother, 44, Other ethnicity, Breast Cancer treatment, long-term recipient)

‘My family are hugely grateful for the charity and didn’t know such an organisation existed until I had contacted the charity.’ (Mother, 35, White, Breast Cancer, long-term recipient)

Others simply knew how important DHM was to the parents and their support of them using it helped validate and encourage their decision.

‘My dad didn’t really get it but he knew how important it was to me and even drove miles to collect some milk one day. It meant so much to me that he had my back through this’ (Mother, 36, White, Medication issues, short-term recipient)

Notably, sometimes using DHM also stopped wider pressure to give formula milk supplements:

‘It stopped my wider family and friends telling me that it ‘wouldn’t hurt’ to just give her formula’. (Mother, 37, White, Premature baby, long-term recipient)

### Negative, unsupported reactions

Some parents experienced negative reactions, either from their partner or wider family. This made a difficult experience even more challenging, leading parents to feel unsupported and let down just when they needed others the most.

‘It was a bit of a taboo subject and I was criticised for wanting to give my baby donor milk, and this left me feeling isolated during a time when I needed my family’s support.’ (Mother, 28, White, Faltering growth, long-term recipient)

‘My partner didn’t understand at first and tried to suggest formula milk would be better and I found this very upsetting. He did come round but I feel like if he had known more about it at the start it would have been much easier for us all.’ (Mother, 29, Asian British Chinese, Premature baby, short-term recipient)

## Discussion

This study explored the experiences of parents who had received screened DHM from a milk bank for their baby and the impact that this had upon their wellbeing. It was clear that receiving DHM not only eased anxieties over infant health and developmental outcomes, particularly for premature infants or if there was a family history of illness or allergy, but impacted more broadly upon parental mental health. Mothers felt listened to, cared for and part of a connected community of women, and fathers often felt relieved at being able to play a role by ‘doing something’ by collecting the DHM. Notably receiving DHM allowed parents to meet their feeding goals regarding their baby having human milk, and this was reported to have a significant protective impact upon maternal mental health in particular. Building upon existing evidence of the significant cost savings of providing human milk to premature infants [11], our findings highlight that extending DHM provision could play a.

potential preventative role in the significant health burden and cost implications of postnatal mental health issues [23].

Focusing on the impact of infant feeding experience upon maternal mental health, previous.

research has shown that mothers who are motivated to breastfeed can experience significant negative emotions of guilt, grief, and loss if they are unable to meet their breastfeeding goals [24], putting them at an increased risk of postnatal depression [25–27]. This was echoed in our study; mothers talked about feeling devastated, anxious and heartbroken at the realisation they could not breastfeed or do so exclusively. For these motivated women this was more than feeling a little sad and needing to adapt; it was a grief reaction. As with many studies exploring breastfeeding difficulties [28, 29], guilt was a common and central emotion to not being able to breastfeed or do so exclusively, despite every mother in the study experiencing medical complications, with many working hard to build their supply. However, for many, being able to access DHM significantly reduced this guilt and grief.

Our findings further extend our understanding of the complex interaction between infant feeding and mental health. We know that maternal anxiety and guilt related to not being able to breastfeed is based around concern for infant health and development. Receiving DHM was attributed to reducing these concerns, particularly when infants were premature or there was a family history of health complications. Mothers described the pride that they felt at managing to secure DHM for their baby despite complications, echoing the pride breastfeeding mothers [30], or those expressing for their premature infants in the neonatal unit have previously described [31]. It also reflects findings of a US study exploring experiences of receiving DHM where mothers felt proud and empowered to be able to give their baby DHM [18].

Our data suggest that this pride and reassurance is not only linked to the act of directly breastfeeding or expressing human milk, but also applies, although potentially sometimes to a lesser degree when mothers have secured DHM for their baby. We raise the proviso of ‘sometimes to a lesser degree’ as some mothers in the study experienced conflicting emotions around another mother feeding their baby when they could not, which has been reflected in previous research [32]. Others expressed guilt over receiving DHM, fearing that other more vulnerable infants were more deserving. Such mixed emotions require specific exploration in a further study.

Notably many mothers highlighted how receiving DHM helped them to process the information that they had a health issue or complication that prevented exclusive or partial breastfeeding. Experiencing significant breastfeeding difficulties can be a lonely experience, exacerbated by feelings of loss at the expectations of a nurturing relationship. It is stressful and has been described as a ‘constant fight’ to overcome difficulties [31]. DHM allowed mothers to step back from this ‘fight’ and start to recover. This reflects findings with mothers of premature infants who found that expressing their milk and being able to feed their baby helped them to heal from a traumatic [33] or premature [34] birth. It also fits into research which describes how breastfeeding mothers experiencing stress or mental health difficulties sometimes feel that they are managing to do ‘something right’ by breastfeeding [30, 35]. Mothers may not be directly breastfeeding, but in sourcing human milk for their infant, they clearly felt they were playing a role in protecting and supporting their baby.

Fathers in the study also highlighted how the experience of being able to play a practical role by arranging and collecting deliveries of DHM helped them to feel like they were doing something positive to help their partner and baby. This may support paternal mental health as previous research with fathers whose partner was experiencing breastfeeding difficulties, found that many reported feeling helpless and like a ‘spare part’, unable to make things better [36, 37]. It may also play a role in further supporting breastfeeding. We know that mothers are more likely to feel empowered, confident and to continue breastfeeding if they feel supported by partners to breastfeed [38]. Further studies are required to explore if the practical support and participation of fathers around advocating for and collecting DHM helps mothers who are working to build their supply or to come to terms with not being able to breastfeed.

Positive emotions related to receiving DHM were not simply about the milk that the baby was receiving. Part of the protective experience for mothers was feeling listened to and supported in their infant feeding plans. No one judged or laughed at their decision to give DHM or questioned its value – all experiences that breastfeeding women have reported [39–41]. Instead, mothers felt heard, validated, and reassured, key components in protecting maternal mental health [42].

Additionally, mothers had the experience of reaching out for support when experiencing a feeding difficulty and receiving the support they needed – a critical aspect of the relationship between breastfeeding and maternal mental health [43, 44]. A lack of support to breastfeed and subsequently needing to stop prematurely can exacerbate breastfeeding grief and postnatal depression [25–27]. The experience of not receiving the support they presumed would occur due to heavy promotion of breastfeeding antenatally can leave mothers feeling let down, gaslit and angry [35, 41, 45]. Simply being listened to and having their wishes respected appeared to have a positive influence on maternal wellbeing separate from receiving the DHM for their baby.

Related to this was the value mothers placed on being able to follow through with their infant feeding plans. Although they experienced a complex array of emotions at not being able to breastfeed fully (or fully at first), many valued their feeding decisions being respected and the option to be able to choose to use DHM over infant formula. The relationship women have with breastfeeding extends far beyond health impacts, including bodily autonomy, and cultural and religious beliefs [45–47]. Breastfeeding success can be closely tied to maternal identity and preferred way of mothering [48, 49]. Our data suggest that this experience may also apply when being able to source DHM in the context of health barriers.

Some mothers reported family and friends not understanding or valuing their decision to use DHM. Some were critical, leaving mothers feeling even more unsupported at a challenging time. Family attitudes to breastfeeding can be complex, with critical or inaccurate views affecting the support new mothers receive. These are often bound up in personal difficult experiences of feeding babies, with some feeling criticised when their own daughter chooses to feed in a different way [50, 51]. Although research in this area has typically focused on breastfeeding, it is likely that similar concepts apply, alongside views around human milk as a potentially contaminated or sexual fluid [52] in contrast to carefully designed ‘scientific’ and ‘sanitised’ adverts of infant formula milk [53] may influence how some people react towards the concept of DHM. Further research needs to explore public perceptions and understanding of DHM and develop public health education programmes.

We did not quantitatively measure mental health in this study but did reflect on duration of DHM use and how this may have affected parental wellbeing. Although the majority of participants received DHM for a week or less, our sample included 26 parents who received DHM for a longer duration. These participants were typically mothers who had longer-term health / medication issues, were undergoing cancer treatment or undergoing a mastectomy – although some received milk alongside building their own supply. Both short- and long-term recipients benefitted from receiving milk, but those receiving milk for a longer duration tended to express a stronger protective impact upon wellbeing and are overrepresented in our quotes based on the sample split. Given DHM use in the UK is predominantly restricted to shorter-term use for premature infants our initial findings her warrant further research into the potential clinical mental health impacts that might arise from greater DHM availability for those undergoing cancer treatment or living with illnesses such as HIV.

Finally, a number of mothers in our study noted that receiving DHM helped them to establish breastfeeding. The literature examining whether DHM supplementation has any impact on continued breastfeeding rates over formula milk supplementation is limited. One review found that DHM increased the rates of any breastfeeding at discharge from hospital but had no effect on exclusive breastfeeding rates [54]. Another study retrospectively examined the effect of DHM as a feeding supplement instead of formula milk in a level 1 NICU on exclusive breastfeeding rates at 6 months. Comparing feeding outcomes at six months for 73 infants who received formula milk supplementation before the implementation to 49 infants who received DHM after, infants who received DHM were five-fold more likely to just be receiving breastmilk at six months old [55].

Our data supports preliminary explanations as to why DHM may protect the breastfeeding relationship more than formula supplementation. DHM enabled their baby to be exclusively human milk fed which increased motivation to continue this – more so than if formula supplementation had been introduced. This echoes findings in another study where mothers saw formula introduction as a more permanent change to their baby’s diet whereas DHM represented a short-term bridge to exclusive breastfeeding [17]. Additionally, some mothers commented that they felt that they needed to continue trying to establish their supply as other women had taken the time to express and donate their milk to support them.

The study was limited in that the sample was self-selecting both in terms of participation and whether DHM was received. It is likely that parents who were already motivated to seek DHM and had a positive experience were more likely to complete the survey meaning that more negative or neutral experiences may not be included. Our sample was also weighted towards older participants with higher levels of education; this represents a common limitation of survey research but also may reflect who is seeking out and using DHM [19]. In terms of ethnicity our sample is similar to the demographic representation in the UK, but given a higher rate or premature birth amongst Black, Asian and Minority Ethnic groups [56] likely under-sampled parents from non-White backgrounds. Finally, although the sample was spread across at least nine milk banks (with some participants unsure) and participants recruited from across the UK, not every UK milk bank was represented, potentially excluding regionalised experiences.

The survey was distributed via social media. Data suggests that social media-based samples tend to be skewed towards more educated, older demographics [57], although further research is now needed to understand how the global Covid-19 pandemic may have affected internet use and participation in online surveys, due to major increases in internet-based activity during lockdowns [58]. It would have been ideal to collect data via all neonatal units or milk banks in the UK to increase sample variability. However, given the timing of data collection during the pandemic and the early stages of this research topic it was felt that this would place an additional burden upon study gatekeepers who at the time of data collection were urgently focussing on ensuring DHM provision was maintained and processes were safe [59].

Additionally, self-selecting samples have a tendency to be weighted towards older mothers with a higher level of education for subjects such as infant feeding research even when hospital cohort studies are used. Our sample reflects similar demographics to studies that have recruited on similar research topics by inviting all eligible participants in a hospital cohort [60–62]. However, caution should always be taken in noting who did not take part and further research may wish to build on this basis using different methods of recruitment. More focussed recruitment techniques may be necessary to encourage and facilitate participation from parents who are typically under-represented in infant feeding studies.

Our decision to include fathers / partners in the research was also important as they may play a key role in DHM provision. However, only small numbers took part and care must be taken when extrapolating from these findings. It is likely that given our method of recruitment that fathers / partners may have been made aware of the study by their partner with both parents’ experiences added to the current data set. Although this provides added depth to the data caution in interpreting findings is needed, with further research needed to understand the experiences of fathers/ partners and how this affects DHM use and acceptability.

Finally, further research needs to measure prospectively any potential link between receiving DHM and anxiety and depression. Work is ongoing to determine whether receiving DHM reduces symptoms, or a broader impact upon perceived mental health. Looking to the longer term it would be useful to understand whether DHM impacts upon mental health only amongst parents who are strongly in favour of its use and motivated to receive it. Future randomised controlled trials will be needed to understand whether access to DHM confers a protective impact for the broader population.

## Conclusions

To conclude, our data suggests that this limited sample of self-selecting parents who have received DHM perceived that such support improved their mental health, made them feel listened to and cared for, and that their infant feeding wishes were respected. Receiving DHM and/or not needing to give their baby formula milk were considered to be protective for mental health and wellbeing by parents who were highly motivated for their baby to receive human milk. Given the significant personal, social and financial impact that postnatal depression has upon parents and communities, our research indicates a further reason to improve the availability of DHM.

## Data Availability

The datasets generated and/or analysed during the current study are not publicly available to protect participant confidentiality but are available in an anonymised form from the corresponding author on reasonable request.
